# Expression of Elongation Factor (EF)-Tu Is Correlated with Prognosis of Gastric Adenocarcinomas

**DOI:** 10.3390/ijms12106645

**Published:** 2011-10-10

**Authors:** Chaoyang Xu, Jianjun Wang, Jiajia Li, Rengui Fang

**Affiliations:** 1 Department of Breast and Thyroid Surgery, Shaoxing People’s Hospital, Shaoxing Hospital of Zhejiang University, Shaoxing, Zhejiang, 312000, China; 2 Department of Surgical Oncology, Sir Run Run Shaw Hospital, College of Medicine, Zhejiang University, Hangzhou, Zhejiang, 310016, China; 3 Department of Surgical Oncology, The Chunan County First Hospital, Chunan, Zhejiang, 311700, China; E-Mails: wjj0714@163.com (J.W.); fang_rengui@yahoo.com.cn (R.F.); 4 Department of Surgery, Shaoxing People’s Hospital, Shaoxing Hospital of Zhejiang University, Shaoxing, Zhejiang, 312000, China; E-Mail: dr_xuchaoyang@yahoo.com.cn (J.L.)

**Keywords:** elongation factor Tu, gastric cancer, survival

## Abstract

Altered expressions of mitochondria elongation factor Tu (EF-Tu) have been observed in certain types of cancers, including gastric cancer cell lines, but the impact of the alterations in gastric adenocarcinoma remains unclear. The purpose of this study was to investigate the expression of EF-Tu in gastric adenocarcinoma and to assess its clinical significance. A total of 104 paired resected gastric adenocarcinoma and corresponding normal specimens were collected in this study. EF-Tu expression was assessed by immunohistochemical staining. The correlation of EF-Tu expression and patients’ clinicopathological parameters was statically evaluated and the prognostic significance of EF-Tu expression was assessed by univariate and multivariate analyses. Forty-nine out of 104 (47.1%) gastric adenocarcinoma specimens showed high expression of EF-Tu, while the remaining 55 specimens showed weak or negative expression of EF-Tu. In contrast, EF-Tu high expression was detected in 62.5% (65 of 104) normal tissues. Down-regulation of EF-Tu was associated with serosal invasion (*P* = 0.042) and node involvement (*P* = 0.005), and down-regulation of EF-Tu was correlated with poor overall survival (*P* = 0.020). In curative resection (R0) patients, there were also significant differences (*P* = 0.043). In the multivariate analysis, the EF-Tu expression remained a significant independent prognostic factor (*P* = 0.038). Our results indicate that EF-Tu is expressed in both gastric adenocarcinoma and corresponding normal tissues. Down-regulation of EF-Tu expression is associated with advanced disease stage and EF-Tu expression maybe served as an independent prognostic factor.

## 1. Introduction

Currently gastric adenocarcinoma remains one of the most common cancers worldwide, and one of the leading causes of cancer-related death in China [1]. Gastric adenocarcinoma encompasses many subtypes with distinct genetic and biological features. Identification of new biological markers to determine the risk of poor prognosis is important for designing treatment strategies [2,3].

Elongation factor Tu (EF-Tu, Tu translation elongation factor, TUFM), one of the most abundant proteins of the mitochondrial, plays a key role in the elongation process of mitochondrial protein biosynthesis [4]. The main function of the translation factor EF-Tu is to deliver aminoacyl-tRNA to the A-site on the ribosome. In addition, other functions and characteristics have been reported for EF-Tu, including its chaperone properties [5,6] and roles in signal transduction [7]. Expressions of EF-Tu have been observed in a variety of tissues with high level of expression in tissues with intrinsically active oxidative metabolism such as heart [8] and brain [9].

Recent studies have shown that the expression level of EF-Tu is altered in several types of cancers. Up-regulation of both EF-Tu and the cytoplasmic elongation factor EF-1a in human pancreatic adenocarcinoma has been reported [10,11], whereas absence of EF-Tu expression in human hepatocellular carcinoma cell lines HepG2 was also observed [12]. These studies yielded variable and, in some cases, contradictive results in terms of the expression of EF-Tu in different tumor cells [11–13]. Furthermore, one recent study showed that EF-Tu was up-regulated in human gastric cancer cell line AGS compared to the rat normal gastric cell line RGM-1, and it was then proposed that EF-Tu might be served as potential cancer biomarkers in mitochondria [14].

The purpose of the present study was to examine the expression status of EF-Tu in gastric adenocarcinoma tissues and to evaluate whether EF-Tu expression level correlates with the clinicopathological parameters and prognosis of gastric adenocarcinoma patients. To the best of our knowledge, this is the first report on evaluating expression of the EF-Tu in clinical specimens of gastric adenocarcinoma. Our results indicate that EF-Tu down-regulation correlates well with advanced disease stage and EF-Tu expression is an independent prognostic factor.

## 2. Materials and Methods

### 2.1. Case Selection

Paired specimens of gastric adenocarcinoma and corresponding normal specimens were obtained from 104 patients who underwent surgical resection of gastric adenocarcinoma in the department of surgical oncology, Sir Run Run Shaw Hospital, Zhejiang University College of Medicine between July 1995 and March 2007, with informed consent under the guideline of Hospital Ethics Committee. Among the 104 patients, 13 patients who had synchronous peritoneal metastasis were included in this study. The patients comprised 68 males and 36 females aged 38 to 88 years old (mean = 67.5 years old). None of the patients received any anti-cancer treatment before surgery. Correlation between expression of EF-Tu and clinicopathologic parameters including age, sex, pTNM pathological classification of the International Union against Cancer (UICC) [12], peritoneal dissemination, and lymph node metastasis were evaluated.

### 2.2. Immunohistochemistry

Immunohistochemical analysis for EF-Tu expression was performed on formalin-fixed, paraffin-embedded sections of surgical specimen. The slides were deparaffinized in xylene and rehydrated in gradient ethanol solutions. Endogenous peroxidase was blocked with 0.3% H_2_O_2_ in methanol for 10 min. The slides were immersed in 10 mM citric buffer (pH 6.0) with heating for 15 min for antigen retrieval. The slides were cooled at room temperature for 20 min and then washed with phosphate buffered saline (PBS). Nonspecific binding was blocked by preincubation with 10% fetal calf serum in PBS with 0.01% sodium azide, and the slides were incubated in a humid chamber for 1 h with antibody against EF-Tu (mouse monoclonal, CBP-KK1, Santa Cruz; 1:200). After washing three times in PBS, the slides were incubated with the EnVision-HRP complex (undiluted, DAKO) for 60 min. The slides were visualized with diaminobenzidine (DAKO Corp., Changzhou, China) and then counterstained with hematoxylin. For substitute negative controls, the primary antibody was replaced with phosphate buffered saline. Positive controls included gastric adenocarcinomas tissue known to exhibit high levels of marker.

All the slides were examined and scored independently by two experienced pathologists to avoid subjective biases. The criteria for EF-Tu expression level was as follows: no cytoplasm staining was given a score 0; faint/barely perceptible cytoplasm staining detected in >25% of tumor cells was scored as 1+; a moderate cytoplasmic staining observed in >25% of tumor cells was scored as 2+, a high cytoplasmic staining observed in >25% of tumor cells was graded 3+ respectively. A score of 0 was considered negative and 1+ was considered weak positive, whereas 2+ and 3+ were considered high positive.

### 2.3. Follow Up

The patients were followed up until death or until the date of last follow-up of 30 November 2007. Thirty-six patients (34.6%) died during the follow-up period, and the median follow-up interval was 50.6 months (range: 3 to 127 months).

### 2.4. Statistical Analysis

All statistical analyses were conducted using the statistical program SPSS 15.0 for windows (SPSS, Chicago, IL, USA). Pretreatment characteristics were analyzed using the 2-tailed chi-square test, and the 2-tailed t test was used to evaluate correlation between EF-Tu expression and clinicopathologic parameters. Univariate analysis of patient survival was performed using Kaplan-Meier method. The survival curves were compared using the log-rank test. Multivariate analyses were done using logistic regression and Cox’s proportional hazard model. The accepted level of significance was set as *P* < 0.05.

## 3. Results

### 3.1. EF-Tu Expression Correlates with Clinicopathologic Parameters

**Figure 1:**
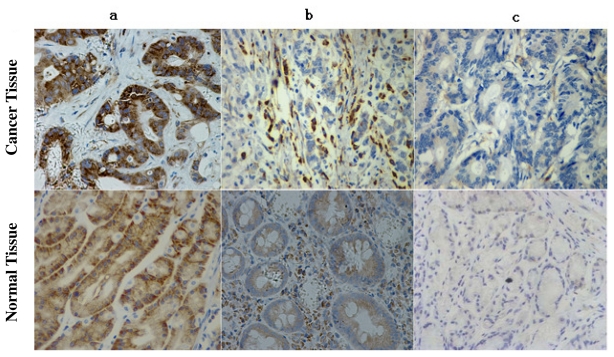
Immunohistochemical analysis of elongation factor Tu (EF-Tu) expression in gastric adenocarcinomas. (**a**) High complete cytoplasmic staining observed in >25% of tumor cells; (**b**) faint/barely perceptible cytoplasmic staining detected in >25% of tumor cells; (**c**) No staining observed.

**Figure 2:**
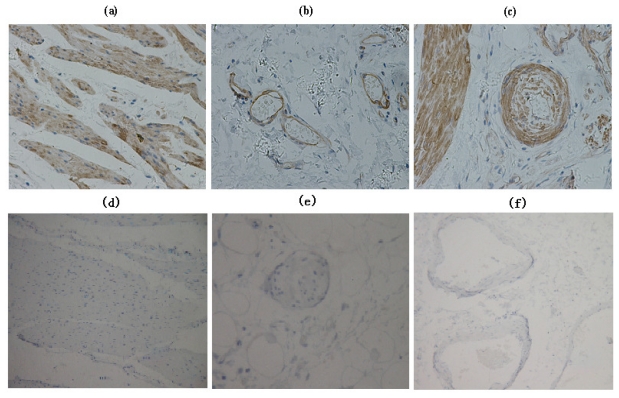
Immunohistochemical analysis of EF-Tu expression in interstitial tissue: (**a**) Smooth muscle; (**b**) venous endothelial cells; (**c**) artery endothelial cells, showed high EF-Tu expression. Immunohistochemical analysis of EF-Tu negative control: (**d**) Smooth muscle; (**e**) venous endothelial cells; (**f**) artery endothelial cells.

The majority (94.2%, 98 of 104) of gastric adenocarcinomas showed positive expression of EF-Tu. In a total of 104 gastric adenocarcinoma specimens, 55 (52.9%) showed high EF-Tu expression, 39 (41.3%) showed weak EF-Tu expression and 6 (5.8%) showed absence of EF-Tu expression (Figure 1). EF-Tu was also observed in 103 (99%) corresponding normal tissues with 65 (62.5%) showed high expression and 38 (36.5%) showed low expression. Lymphocytes, vascular endothelial cells, and smooth muscle also showed high EF-Tu expression (Figure 2).

EF-Tu expression was correlated with depth of invasion, and node involvement. However, no difference was found between both groups regarding sex, age, tumor differentiation, location, type of operation, ecog, tumor size, distant metastasis (Tables 1 and 2).

### 3.2. Univariate Analysis

**Figure 3:**
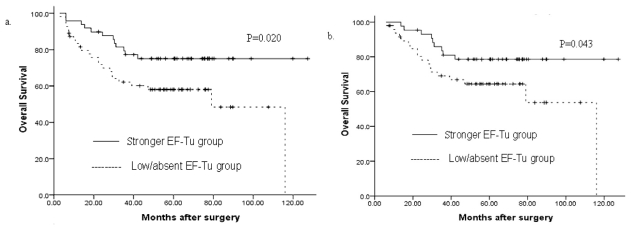
(**a**) The mean overall survival time for the negative/weak EF-Tu expression group was 71.6 months, and for the high EF-Tu expression group was 101.7 months (*P* = 0.020); (**b**) In R0 (radical operation) gastric cancer, the mean overall survival time for the negative/weak EF-Tu expression group was 79.0 months and for the high EF-Tu expression group was106.2 months (*P* = 0.043).

In univariate analysis tumor location, tumor size, node invasion, distant metastasis, type of operation and EF-Tu expression were associated with overall survival. Based on the expression level of EF-Tu, the mean overall survival time for negative/low EF-Tu expression group were 71.6 months and for high EF-Tu expression group, 101.7 months, respectively. The difference in survival was significant (*P* = 0.020) (Figure 3a). Even in the curative resection cases, a mean overall survival time for negative/weak EF-Tu expression group were 79.0 months, and for high EF-Tu expression group 106.2 months respectively. The difference in survival was also significant (*P* = 0.043) (Figure 3b). These data suggest down-regulated EF-Tu expression correlates with poor prognosis.

### 3.3. Multivariate Analysis

The following significant parameters were entered into a multivariate analysis: EF-Tu, distant metastasis, serosal invasion, number of mLNs, number of tLNs, type of operation, age, tumor size, histological type, gender, tumor location. In terms of both overall and recurrence-free survivals, the EF-Tu expression was the independent predictor (hazard ratio 0.460, *P* = 0.038 Table 3), which suggests EF-Tu expression served as an independent prognostic factor.

## 4. Discussion

Elongation factor Tu (EF-Tu) is involved in mitochondrial protein synthesis, which complexes with GTP and transports the aa-tRNA to the programmed ribosome [15,16]. Moreover, it can participate in other cellular processes such as organization of mitotic apparatus, developmental regulation, aging, cell morphology and transformation [17–19]. Recent studies have shown that dysregulation of EF-Tu is involved in the oncogenic process [14]. However, both decreased and increased levels of EF-Tu expression have been found in different human cancers, and these contradictory results led to different conclusions for the roles of EF-Tu in carcinogenesis. In hepatocellular carcinoma, down-regulation of EF-Tu expression was found in cell line HepG2, while up-regulation was found in another cell line HCC-S102 [12]. In addition, EF-Tu was up-regulated in human gastric cancer cell line AGS compared to the rat normal gastric cell line RGM-1 by two-dimensional electrophoresis proteomic analysis on the mitochondria-enriched fractions, which may influence the function of the mitochondrial [14]. In our preliminary study, we also observed different levels of EF-Tu expression in some tumor cell lines, including high level EF-Tu expression in breast cancer cell line MCF7, leukemia cell line k562 and gastric cancer cell lines SGC-7901, MKN-45 and KatoIII, and low level EF-Tu expression in gastric cancer cell lines MKN-28 and MKN-74. All these investigations indicate that the role EF-Tu plays in the oncogenic process, may be very complicated.

In the current study, we examined EF-Tu expression in 104 gastric adenocarcinoma cases and showed that EF-Tu staining in specimens is predominantly localized on the cell membrane/cytoplasm, and the level of EF-Tu expression decreased in tumor specimens as compared to normal counterparts. Down-regulation of EF-Tu expression in tumor specimens showed a powerful adverse prognostic effect in our study. The effect of EF-Tu expression status was independent of other known predictors, such as the nodal ratio, tumor size, depth of invasion or distant metastasis. Furthermore, EF-Tu expression level was associated with curative resection (R0) patients’ outcome. The interesting thing was that all six EF-Tu-negative patients recurred after operation. To increase case numbers in future may provide more clear and solid evidence.

Although there have been several studies on EF-Tu expression in different cancer cell lines [20–22], no investigation on EF-Tu expression in gastric adenocarcinoma specimens and correlation analysis between EF-Tu and adenocarcinoma patients’ clinicopathological parameters and prognosis has been carried out to date. Recent molecular-profiling studies have identified EF-Tu dysregulation as a potentially important event in cancer development and proposed it may serve as potential cancer biomarkers in mechanism of the mitochondrial changes in gastric tumor cell [14], but this postulation lacks clinical evidence. It is not yet clear how EF-Tu expression level change affects the survival of patients with gastric adenocarcinoma. Since EF-Tu displays chaperone activity in the quality control of misfolded newly synthesized polypeptides in mitochondria in a GTP-independent manner [23,24], we speculated that when EF-Tu was down-regulation, the peptides synthesized from mitochondria ribosomes may be misfolded, which may lead to tumor development and/or progression [25,26].

Mitochondria are key players in several cellular functions including growth, division, energy metabolism and apoptosis, and its dysfunction contributes to a number of human disorders and may aid cancer progression [27]. It is believed that gastric adenocarcinoma is associated with a severe hypoxia and mitochondria degeneration, when comparing with normal subjects [28,29]. EF-Tu participates in the elongation process of protein biosynthesis in mitochondria, and down-regulation of EF-Tu may suggest the dysfunctions of mitochondria, which might be another reason for poor survival in patients with EF-Tu down-regulation.

One of the main characteristics of cancer cells is their rapid proliferation and this easily causes local hypoxic environment, due to the inability of the local vasculature to supply an adequate amount of oxygen. Because of the inability of mitochondria to provide enough ATP for cell survival under hypoxic conditions, tumor cells must up-regulate the glycolytic pathway [30,31]. This occurs by induction of hypoxia-inducible factor 1 (HIF-1) which also suppresses mitochondrial function in tumor cells [32,33], suggesting a hypoxic relationship with mitochondrial dysfunction.

In summary, we have shown that expression of EF-Tu can be used as a potential molecular marker to predict patient outcome in gastric adenocarcinoma patients. Our data showed that negative or low EF-Tu expression has a high correlation with poor survival both in univariate and multivariate analyses. Therefore future investigation on elucidation of the precise roles of EF-Tu expression in gastric adenocarcinoma will provide new insights, which will contribute to improved diagnosis and treatment.

## Figures and Tables

**Table 1:** Comparison of demographic and perioperative data in gastric adenocarcinoma patients.

	Negative/low (*n* = 55)	High (*n* = 49)	*P* value
Age	54.9 ± 11.3	57.2 ± 12.5	0.329
Gender			0.104
Male	40 (61.5%)	28 (51.7)	
Female	15 (38.5%)	21 (48.3)	
ECOG			0.544
0	18 (32.7)	17 (35.4)	
1	37 (67.3)	31 (62.5)	
2	0	1 (2.1)	
Type of operation			1.000
Total gastrectomy	6 (10.9)	5 (10.2)	
Subtotal gastrectomy	49 (89.1)	44 (89.8)	

ECOG: Eastern Cooperative Oncology Group; age represents as mean ± SD; values in the parenthesis are percentage.

**Table 2:** Comparison of clinicopathologic features in gastric adenocarcinoma patients.

	Negative/low (*n* = 55)	High (*n* = 49)	*P* value
**Location**			0.753
Upper body or whole	13 (23.6)	9 (18.5)	
Lower or middle body	42 (76.4)	40 (81.5)	
**Tumor differentiated**			0.061
Well	7 (12.7)	2 (4.1)	
Moderate	14 (25.5)	22 (40.0)	
Poor	34 (61.8)	25 (55.9)	
Tumor size	5.3 ± 2.3	4.3 ± 2.0	0.021
**Serosal invasion**			0.042
No	14 (25.5)	22 (44.9)	
Yes	41 (74.5)	27 (55.1)	
**Node involveme**			0.005
No	13 (23.6)	25 (51.0)	
Yes	42 (76.4)	24 (49.0)	
Mean no. of tLNs	21	19	0.489
**Distant metastasis**			1.000
No	48 (87.3)	43 (23.6)	
Yes	7 (12.7)	6 (23.6)	
**Normal specimens EF-Tu expression**			0.015
Negative/low	27 (49.1)	12 (24.5)	
High	28 (50.9)	37 (75.5)	

tLNs indicates total retrieved Lymph Nodes; LN: Lymph Node.

**Table 3:** Results of Stepwise multivariate analysis for Prognostic Factors (*n* = 104). Values in parentheses are 95% confidence intervals. * Cox proportional hazards model.

Variable	HR	95% CI	*P* * value
EF-Tu	0.460	0.220–0.960	0.038
Distant metastasis	4.323	1.442–12.963	0.009
Serosal invasion (yes *vs.* no)	6.242	1.808–21.548	0.004
mLNs	1.082	1.001–1.169	0.047
tLNs			0.053
Type of operation			0.096
Age (years) (>55 *vs* ≤55)			0.187
Tumor size (≦4 cm *vs* >4 cm)			0.547
Histological type (undifferentiated *vs* differentiated)			0.812
Gender			0.810
Tumor location			0.856

HR indicates hazards ratio; 95% CI, 95% confidence interval; mLNs: metastatic lymph nodes; tLNs: total retrieved lymph nodes; Variables that were entered into the regression model were EF-Tu, distant metastasis, Serosal invasion, mLNs, tLNs, type of operation, age, tumor size, histological type, Gender, tumor location. Number of mLNs, and number of tLNs were consecutive variables; others were categorized variables.
